# Targeted Next-Generation Sequencing of Plasma DNA from Cancer Patients: Factors Influencing Consistency with Tumour DNA and Prospective Investigation of Its Utility for Diagnosis

**DOI:** 10.1371/journal.pone.0162809

**Published:** 2016-09-14

**Authors:** Pamela J. Kaisaki, Anthony Cutts, Niko Popitsch, Carme Camps, Melissa M. Pentony, Gareth Wilson, Suzanne Page, Kulvinder Kaur, Dimitris Vavoulis, Shirley Henderson, Avinash Gupta, Mark R. Middleton, Ioannis Karydis, Denis C. Talbot, Anna Schuh, Jenny C. Taylor

**Affiliations:** 1 The Wellcome Trust Centre for Human Genetics, University of Oxford, Oxford, United Kingdom; 2 National Institute for Health Research Oxford Biomedical Research Centre (NIHR Oxford BRC), Oxford, United Kingdom; 3 Biomedical Research Centre/National Health Service Translational Molecular Diagnostics Centre, Oxford University Hospitals, John Radcliffe Hospital, Oxford, United Kingdom; 4 Department of Oncology, University of Oxford, Churchill Hospital, Oxford, United Kingdom; CNR, ITALY

## Abstract

Use of circulating tumour DNA (ctDNA) as a liquid biopsy has been proposed for potential identification and monitoring of solid tumours. We investigate a next-generation sequencing approach for mutation detection in ctDNA in two related studies using a targeted panel. The first study was retrospective, using blood samples taken from melanoma patients at diverse timepoints before or after treatment, aiming to evaluate correlation between mutations identified in biopsy and ctDNA, and to acquire a first impression of influencing factors. We found good concordance between ctDNA and tumour mutations of melanoma patients when blood samples were collected within one year of biopsy or before treatment. In contrast, when ctDNA was sequenced after targeted treatment in melanoma, mutations were no longer found in 9 out of 10 patients, suggesting the method might be useful for detecting treatment response. Building on these findings, we focused the second study on ctDNA obtained before biopsy in lung patients, i.e. when a tentative diagnosis of lung cancer had been made, but no treatment had started. The main objective of this prospective study was to evaluate use of ctDNA in diagnosis, investigating the concordance of biopsy and ctDNA-derived mutation detection. Here we also found positive correlation between diagnostic lung biopsy results and pre-biopsy ctDNA sequencing, providing support for using ctDNA as a cost-effective, non-invasive solution when the tumour is inaccessible or when biopsy poses significant risk to the patient.

## Introduction

Cell-free DNA (cfDNA) has been known to exist in blood since 1948, and is present in all people to some degree[1,2]. In maternal plasma, cfDNA has proven to be a useful source of fetal genetic material to diagnose certain inherited conditions during pregnancy [3]. In cancer patients, solid tumours often release DNA into the circulation, leading to the suggestion that blood-derived circulating tumour DNA (ctDNA) could be a source of “liquid biopsy” and act as a surrogate for traditional tumour biopsy [4]. Because it helps to establish the mutational spectrum of the tumour, analysis of ctDNA has been reported to be of potential value in determining prognosis, monitoring tumour evolution during treatment and detection of relapse [5, 6]. Concordance between tumour and plasma DNA mutations has been reported by several groups, in early and late-stage cancers [7–9].

It is well-documented that solid tumours are not composed of a single oncogenic clone, but have extensive inter- and intra-tumoural genetic heterogeneity [10]. This variation is due in part to genomic instability caused by defects in DNA repair and replication, and in part due to the effects of treatment, when new driver mutations may emerge as treatment-sensitive clones diminish. Next-generation sequencing (NGS) of a targeted panel of cancer genes allows simultaneous detection of a large set of informative mutations from small amounts of material that are usually taken from a single biopsy. Theoretically, ctDNA should be more representative of the spatial heterogeneity of the tumour compared with discrete biopsies. Lung cancer and melanoma exhibit the highest frequency of somatic mutations of the solid cancers and present the greatest opportunities for targeted and personalised therapies.

Previous NGS analysis of ctDNA has detected a wide range of mutant allele frequencies, from 52% [11] down to 2–3% [4, 6]. Structural variants and copy number variations have been measured down to levels of 0.75–0.9% [11, 12]. Other approaches, which rely on the capture of specific recurrent mutations before sequencing, can detect allele frequencies as low as 0.02% [13].

Here we study two aspects of next-generation sequencing in ctDNA: first we identify factors influencing the concordance between mutations in melanoma tumours and circulating DNA. Second, we examine whether ctDNA sequencing can be used in lung cancer diagnosis, by sequencing plasma DNA taken prior to bronchoscopy.

## Materials and Methods

### Patient samples

This study, under the authority of the Oxford Radcliffe Biobank (ORB), was reviewed and approved by the South Central—Oxford C Research Ethics Committee (REC reference number 09/H0606/5+5), before the study began. Written informed consent was provided by participants (melanoma and suspected lung cancer patients) according to current ORB guidelines. Blood was drawn, stored at room temperature and processed within 6 hours. In the case of lung patients, blood samples were taken a few minutes prior to tumour biopsy.

### Sample processing and extraction of circulating nucleic acids

In order to minimise lymphocyte lysis, blood samples were centrifuged at 2060 x g (3000 rpm in Beckman GS-6R centrifuge) for 10 minutes at room temperature without brake within 6 hours of collection [14]. Plasma was transferred to a new tube, mixed, and aliquots were pipetted into microfuge tubes. After 10 min in the centrifuge at 7000 rpm, the supernatants were transferred to new microfuge tubes and stored at -80°C until DNA extraction. Plasma DNA was isolated using Qiagen QIAamp Circulating Nucleic Acid kit according to the manufacturer’s protocol (QIAGEN Ltd., Manchester, UK). Matching gDNA was extracted from whole blood using Qiagen QIAamp DSP DNA Blood mini kit. DNA quantity was determined using Qubit dsDNA High Sensitivity assay kit on a Qubit 2.0 Fluorometer (ThermoFisher, Paisley, UK).

### Preparation of AmpliSeq libraries for sequencing on the Ion Torrent^™^ PGM^®^

Sequencing libraries were prepared with the Ion Ampliseq Cancer Hotspot Panel. The panel contains a collection of primers designed to interrogate hotspot regions in genes commonly mutated in cancer. Over the course of this study, the first version of the panel, which targeted 46 genes, was replaced by a new version targeting 50 genes (https://tools.lifetechnologies.com/content/sfs/brochures/Ion-AmpliSeq-Cancer-Hotspot-Panel-Flyer.pdf, ThermoFisher). Libraries were generated according to the manufacturer's protocol. Briefly, multiplex PCR was performed with the Ion Ampliseq library kit 2.0 using approximately 10 ng DNA and the panel primer pool. IonXpress-barcoded adapters were attached to the amplicons by ligation. The libraries were purified using Agencourt AMPure XP magnetic beads (Beckman Coulter Ltd, High Wycombe, UK) and either quantified by qPCR using the Ion Library Quantitation Kit (melanoma samples) or amplified using adapter primers (lung samples). Libraries quantified by qPCR were diluted 1:100 and run in duplicate on an ABI 7500. Amplified libraries were purified again, and then quantified on an Agilent 2100 Bioanalyzer using the High Sensitivity DNA kit (Agilent Technologies, Stockport, UK). Library templates for sequencing were prepared by emulsion PCR on the One Touch 2 instrument and loaded onto 318 semiconductor chips and the Ion Torrent PGM^™^ sequencer [15].

### Sequence analysis

The analysis was run with Torrent Variant Caller (TVC) v4.4.5, which was reported to be capable of detecting variants at frequencies of 0.5% [16]. We ran a sensitivity study with spiked-in mutation positive controls and confirmed that the detection limit of 0.5% was a suitable threshold (S1 File, S1 Fig, S1 Table). TVC was called in hotspot mode with 5 inputs: a BAM file, a fasta file of hg19 genome reference, a BED file marking targeted regions, a VCF file specifying hotspot locations, and a parameter file. This TVC version and accompanying parameter set have been optimised to detect single nucleotide polymorphisms (SNPs), hotspot insertion-deletions (indels) smaller than 10bp and non-hotspot indels of at least 5 bp and has been validated with over 400 variants (390 SNP, 15 indel).

Pearson’s product-moment correlation coefficients were computed using the R function ‘cor’. Permutation-adjusted (n = 10000) p-values were calculated using a two-tailed test in the R function ‘cor.test’.

### Competitive Allele-Specific TaqMan PCR (castPCR)

Competitive Allele-Specific TaqMan^®^ PCR (castPCR^™^) (ThermoFisher) is a quantitative PCR method that detects specific known mutations [17]. A reference assay is included that measures the total amount of the gene. Nine castPCR assays were available for variants we detected by the cancer hotspot panel, and these were tested on plasma DNA as an orthogonal method. Assays were performed using a BioRad CFX96 Real-Time PCR Detection System. Percent mutation was calculated as follows:

$$\%\text{ Mutation}=2^{\text{-dCt}}*100\%$$

Where dCt (delta Ct) = Ct mutant–Ct reference (Ct is the threshold cycle, where the fluorescent signal crosses a significant threshold determined automatically by the BioRad CFX96).

## Results

### Good concordance between melanoma ctDNA and primary tumours when samples were collected before treatment or less than one year apart

**Fig 1:**
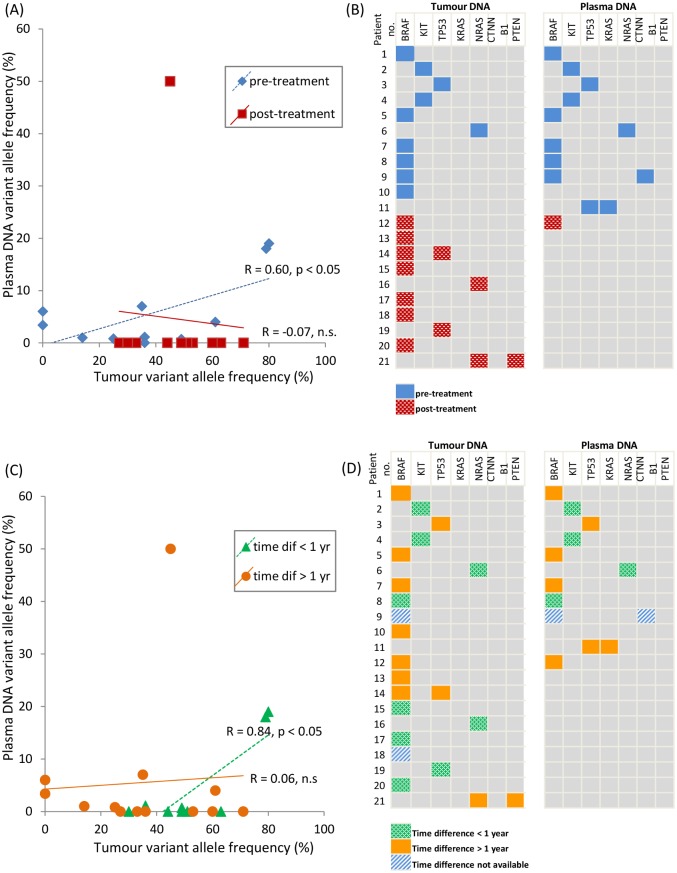
Factors affecting correlation between tumour and plasma DNA variants in melanoma patients. Tumour and plasma DNA from melanoma patients were sequenced using Ampliseq Cancer Hotspot Panel (8–10 samples per 318 chip), and variant results were compared in relation to two factors, pre- or post-treatment sampling and time difference between sampling of tumour and plasma. (A) Pre- or post-treatment correlation. Blood samples taken before treatment are denoted by blue diamonds, dashed line, and samples taken post-treatment are shown by red squares, solid line; (B) Mutated genes in tumour and plasma DNA, marked in blue if plasma taken pre-treatment, red pattern if plasma was post-treatment; (C) Time difference correlation. Blood samples taken less than a year after biopsy are plotted as green triangles, dashed line, and samples taken more than a year after biopsy are shown as orange circles, solid line; (D) Mutated genes in tumour and plasma DNA, marked in green pattern if time difference between biopsy and blood sampling < 1 year, solid orange if time difference > 1 year (tumour biopsy date not available for patients 9 and 18, marked in blue stripe).

This study was a retrospective pilot investigation in melanoma patients to confirm that mutations discovered in the primary tumour were also detectable in ctDNA using the Ion Torrent^™^ platform, and to examine factors that may affect concordance of mutation detection (Fig 1). Patients in this cohort had a confirmed diagnosis of melanoma and their blood samples were collected either before or after treatment.

Fig 1, panels A and B, show the effect of treatment time frame on correlation of variant allele frequency between tumour and plasma DNA. Of eleven patients where blood sampling took place before treatment, nine had the same mutation in both ctDNA and primary tumour, and we found a significant positive relationship between the tumour and ctDNA variant allele frequency, Pearsons R(9) = 0.60, p < 0.05 (Panel A, blue diamonds; panel B, solid blue boxes). In contrast, no correlation with tumour variant allele frequency was observed in the ten patients where plasma DNA sampled after treatment took place (Panel A, red squares; Pearsons R(9) = -0.07, n.s.; panel B, red pattern). In patient 9’s plasma DNA (pre-treatment), we detected an additional mutation (*CTNNB1 T41A*) to the one found in the tumour. In post-treatment samples, the nearly complete absence of mutational findings could be an indication of patients responding to treatment given after tumour biopsy but prior to blood collection for ctDNA extraction. In particular, six of the patients (numbers 13–15, 17, 18, 20) with no mutations detected in ctDNA had previously been given *BRAF* inhibitors Vemurafenib and Dabrafenib (S2 Table). Patient 12 still carried a *BRAF* mutation in ctDNA following treatment.

Fig 1, panels C and D, show the effect of a time lag between sampling of tumour and plasma DNA on correlation of mutation allele frequency between the two, in the same set of patients. When blood samples were taken less than a year after biopsy, ctDNA variant allele frequency is significantly correlated with tumour variant allele frequency (Panel C, green dashed line; Pearsons R (7) = 0.84, p < 0.05; panel D, green pattern boxes), whereas samples taken more than a year after biopsy showed no correlation with the tumour (orange solid line; R (11) = 0.06, n.s., and solid orange boxes). It is interesting that no new mutations arose in these patients in the time frame between treatment and sampling, with the exception of patient 11 in which two mutations were found in ctDNA, but none in tumour. S2 Table shows that patient 11’s KRAS G12R ctDNA mutation was present at 6% allele frequency (172/2843 read counts) and TP53 R248Q at 3.4% (388/11654 read counts), which are comfortably above the detection limit of 0.5%. The lack of mutations found in the tumour could result from biopsy sampling of genomically heterogeneous tumour regions or from tumour evolution during the time lag, which was more than two years between tumour and plasma sampling. The depth of coverage of the tumour sequence at the mutation position was 3454 reads for KRAS and 5930 reads for TP53, respectively, and it is highly unlikely with this deep coverage to be due to lack of detection.

Together, this pilot study with melanoma patients demonstrates that ctDNA could be a reliable surrogate for tumour biopsy when sampled in a similar time frame and before treatment. Although it was a retrospective study, the dearth of mutations detected post-treatment suggests that ctDNA sequencing could be an attainable and easy measure of whether patients are responding to therapy.

### Prospective study in suspected lung cancer patients confirmed high level of concordance between mutations found in ctDNA and diagnostic biopsy

Based on results of the first study, our second study focused on collecting blood samples prospectively from suspected lung cancer patients just prior to diagnostic tumour biopsy (endobronchial or endoscopic ultrasound-guided biopsy). The advantage of this is that ctDNA could be sampled at the same time as the tumour biopsy and would be free from any potential contamination by tumour DNA released into the blood as a consequence of the procedure. Furthermore, because most patients were at relatively early stages in the cancer pathway and were treatment-naïve, the collected ctDNA would preserve the original mutational load and allelic frequencies. Among lung cancer patients, there were seven adenocarcinomas, three squamous carcinomas, one carcinoid tumour, and one small cell lung cancer. Sequencing of ctDNA from 12 lung cancer patients identified 22 non-synonymous variants (Table 1), with some of the mutations predicted or known to affect protein function and some of which are actionable, as annotated in the Jackson Laboratory Clinical Knowledgebase, https://ckb.jax.org/ [18] and ClinVar database, http://www.ncbi.nlm.nih.gov/clinvar/ [19].

**Table 1:** Lung cancer ctDNA sequencing.

ID	Histology	Somatic mutation	Variant	Tumour VAF %	ctDNA VAF%	JAX Clinical Knowledgebase (CKB)^2^ and ClinVar annotation	Implications for Treatment and CKB Reference Link
1178	Adeno^1^	yes	*TP53 R273C*	19	6.8	CKB: Hotspot mutation in DNA-binding domain of TP53 (PMID: 22713868); ClinVar: probable pathogenic	Treatment approach: p53 activator, p53 gene therapy (gene-associated clinical trials available) https://ckb.jax.org/geneVariant/show?geneVariantId=3795
		no	*MET N375S*	37	44	CKB: Lies in extracellular Sema ligand-binding domain, predicted loss of function (PMID: 19723643); ClinVar: benign/ likely benign	May confer resistance to MET targeted agents (PMID: 19723643) https://ckb.jax.org/geneVariant/show?geneVariantId=3356
1530	Metastatic adeno (brain)	no	*MET R988C*	49	50	CKB: Gain of function; no increase in MET phosphorylation, but increased cellular protein phosphorylation and increased proliferation and migration of cultured cells (PMID: 14559814, 20670955, 22973954); ClinVar: conflicting: likely benign(2), uncertain sig(2)	Treatment approach: MET inhibitor (Gene-associated clinical trials available) https://ckb.jax.org/geneVariant/show?geneVariantId=706
		yes	*MET H1112Y*	26	6	CKB: Gain of function; causes constitutive MET phosphorylation and activation of downstream signaling, and transforming in cell culture (PMID: 15064724, 24061647); not found in ClinVar	Treatment approach: MET inhibitor (Gene-associated clinical trials available) https://ckb.jax.org/geneVariant/show?geneVariantId=1004
		yes	*KRAS G12C*	41	8	CKB: Hotspot mutation, inhibits GTPase activity of KRAS leading to increased activation of downstream signaling pathways promoting tumour formation (PMID: 16051643); ClinVar: pathogenic	Confers resistance to EGFR tyrosine kinase inhibitors; Treatment approach: Pan-MEK inhibitor, Pan-PI3K inhibitor, RAS inhibitor (gene-associated clinical trials ongoing) https://ckb.jax.org/geneVariant/show?geneVariantId=979
		yes	*SMAD4 R361H*	34	7	CKB: Hotspot residue in MH2 domain of SMAD4, with predicted loss of function (PMID: 21763698); ClinVar: pathogenic	Rare in lung cancer and for which there is little evidence for targeted therapies https://ckb.jax.org/geneVariant/show?geneVariantId=1780
1533	Metastatic adeno	yes	*BRAF D594G*	17	2	CKB: Mutation impairs BRAF kinase activity but paradoxically activates MEK and ERK through CRAF transactivation (PMID: 20141835); ClinVar: pathogenic	Results in BRAF inactivation and insensitivity to BRAF inhibitors; Treatment approach: MEK1, MEK2 and pan-MEK inhibitors https://ckb.jax.org/geneVariant/show?geneVariantId=897
		yes	*KIT G510C*	21	2	Not found in CKB or ClinVar	none
		yes	*TP53 G244C*	24	3	Not found in CKB or ClinVar	none
594	Adeno	no	*KIT V532I*	50	52	Not found in CKB or ClinVar	none
		no	*MET T1010I*	39	48	CKB: Conflicting reports: increase in MET phosphorylation (PMID: 25605252), or no effect (PMID: 20670955); ClinVar: non-pathogenic	none; https://ckb.jax.org/geneVariant/show?geneVariantId=1388
		yes	*TP53 V172F*	17	3	CKB: Mutation in DNA-binding region of TP53 but uncharacterised so its effect is unknown; not found in ClinVar	none; https://ckb.jax.org/geneVariant/show?geneVariantId=17312
591	Metastatic adeno	yes	*TP53 R249S*	11	0.2	CKB: Hotspot mutation in DNA-binding domain of TP53 (PMID: 22713868), decreased transactivation activity of TP53, and context-dependent transforming ability in cell culture (PMID: 20212049, PMID: 20538734); ClinVar: non-pathogenic	Treatment approach: p53 activator, p53 gene therapy (gene-associated clinical trials available) https://ckb.jax.org/geneVariant/show?geneVariantId=3231
		no	*JAK3 V722I*	47	54	CKB: Mutation in protein kinase 1 domain of JAK3, confers gain of function and activation of JAK3/STAT3 pathway (PMID: 23689514); ClinVar: no information	Treatment approach: Pan-JAK inhibitor or JAK3 inhibitor https://ckb.jax.org/geneVariant/show?geneVariantId=1066
590	Adeno	yes	*CTNNB1 G34V*	22	not found	CKB: Mutation within ubiquitination recognition motif of CTNNB1 (PMID: 15064718), gain of function due to nuclear accumulation of CTNNB1 in liver cancer (PMID: 9671767); ClinVar: conflicting: pathogenic(1); uncertain sig(1)	Treatment approach: CTNNB1 inhibitor, PDPK1 inhibitor, Tankyrase inhibitor https://ckb.jax.org/geneVariant/show?geneVariantId=3973
572	Adeno		negative				
463	Small cell lung cancer	yes	*TP53 G245D*	not avail.	4	CKB: Hotspot mutation in DNA-binding domain of TP53 (PMID: 22713868), decreased activation of p21, and also confers a gain-of-function (PMID: 22214764); ClinVar: pathogenic	Treatment approach: p53 activator, p53 gene therapy (gene-associated clinical trials available) https://ckb.jax.org/geneVariant/show?geneVariantId=4658
		no	*TP53 M237I*		9	CKB: Mutation in DNA-binding domain of TP53 (PMID: 22713868), decreased TP53 transactivation activity in cell culture (PMID: 16492679); ClinVar: pathogenic/likely pathogenic	https://ckb.jax.org/geneVariant/show?geneVariantId=16637
593	Squamous cell	yes	*CDKN2A C72S*	not avail.	17	Not found in CKB or ClinVar	none
		yes	PTEN S59*		21	CKB: Results in premature truncation of PTEN protein, predicted loss of function (UniProt.org); not found in ClinVar	Treatment approach: Pan-AKT inhibitor, AKT1 inhibitor, AKT2 inhibitor, AKT3 inhibitor, Pan-PI3K inhibitor (gene-associated clinical trials available) https://ckb.jax.org/geneVariant/show?geneVariantId=4433
		yes	*TP53 M237I*		28	CKB: Mutation in DNA-binding domain of TP53 (PMID: 22713868), decreased TP53 transactivation activity in cell culture (PMID: 16492679); ClinVar: pathogenic/likely pathogenic	https://ckb.jax.org/geneVariant/show?geneVariantId=16637
		no	*TP53 R175H*		2	CKB: Hotspot mutation in DNA-binding domain of TP53 (PMID: 22713868), decreased activation of *TP53* targets, also confers gain of function to TP53, with aberrant activation of gene transcription (PMID: 10713666, 22114072); ClinVar: pathogenic	Treatment approach: p53 activator, p53 gene therapy (gene-associated clinical trials available) https://ckb.jax.org/geneVariant/show?geneVariantId=735
466	Squamous cell	yes	*TP53 R282W*	not avail.	2	CKB: Hotspot mutation in DNA-binding domain of TP53 (PMID: 22713868), decreased activation of TP53 targets, inhibited AMPK signaling, and promoted tumour development in mouse models (PMID: 24857548); ClinVar: conflicting: likely benign(2); pathogenic(2)	Treatment approach: p53 activator, p53 gene therapy (gene-associated clinical trials available) https://ckb.jax.org/geneVariant/show?geneVariantId=4744
538	Squamous cell	yes	*TP53 Y220C*	not avail.	0.6	CKB: Hotspot mutation in DNA-binding domain of TP53 (PMID: 17401432), loss of function, decreased TP53 transcriptional activity in cell culture (PMID: 16861262, 23630318); ClinVar: pathogenic	Treatment approach: p53 activator, p53 gene therapy (gene-associated clinical trials available) https://ckb.jax.org/geneVariant/show?geneVariantId=980
462	Carcinoid tumour		negative	not avail.	negative		

Blood samples were drawn from patients prior to bronchoscopy. Plasma DNA and genomic DNA from each patient were sequenced using Ampliseq Cancer Hotspot Panel v2, using one plasma DNA:gDNA paired sample per 318 chip. Tumour DNA was sequenced when enough bronchoscopy material was available.

^1^Adeno, adenocarcinoma.

^2^CKB website content is for educational and research purposes only.

In the ctDNA of lung adenocarcinoma patients, we found mutations in *TP53*, *MET*, *KRAS*, *SMAD4*, *BRAF*, *KIT*, and *JAK3* (Table 1). Squamous carcinoma patient ctDNA had several mutations in *TP53*, as well as in *CDKN2A* and *PTEN*. The small cell lung cancer patient plasma DNA had a hotspot mutation in *TP53 (G245D)*. There is significant positive correlation between lung tumour and ctDNA variant allele frequency, Pearsons R(13) = 0.85, p = 0.001.

**Fig 2:**
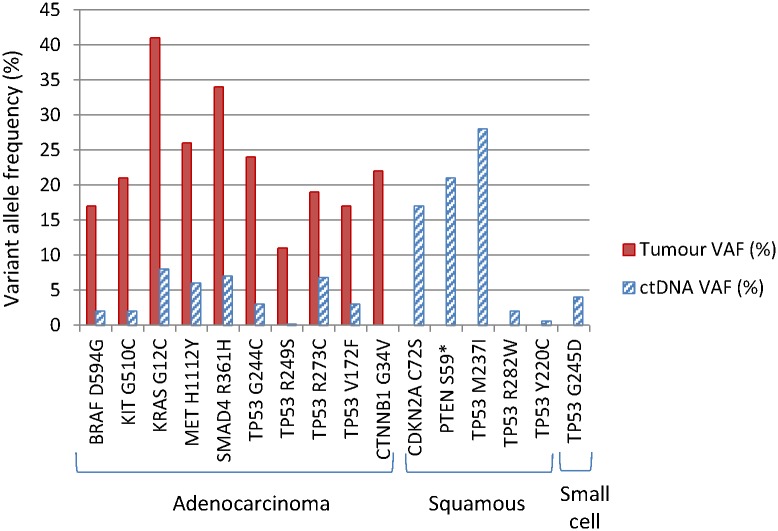
Somatic variants in lung cancer tumour and plasma DNA. Variant allele frequency of mutations are shown in solid red bars for tumour and in hatched blue bars for ctDNA, for different types of lung cancer (Adenocarcinoma; Squamous, squamous cell carcinoma; Small cell, small cell lung cancer). Tumour DNA from squamous and small cell lung cancer patients was not available for sequencing. Mutations are denoted as somatic because they were not present in germline DNA from the same patients.

Fig 2 shows the somatic variants in patients with different types of lung cancer. Sequencing ctDNA confirmed eight out of ten somatic mutations identified from tumour biopsies, and was negative where the tumour was negative. The two remaining mutations were either absent (*CTNNB1 G34V*, no reads detected) or not present in sufficient read counts for detection (*TP53 R249S*, could only be detected at <0.5% by looking manually in Integrative Genomics Viewer (IGV) traces, as listed in Table 1). This suggests that plasma DNA sequencing could be a valuable first-line approach to confirm the diagnosis of lung cancer, but negative findings probably need to be backed up with a biopsy. An additional five patients who were later found not to have cancer, had variants that are annotated as either non-pathogenic in ClinVar database, common SNPs in UCSC genome browser, synonymous variants, or not characterised (S3 Table). All the variants in S3 Table were also found in the patient’s germline DNA (i.e. were not somatic changes).

We attempted to validate the variants identified in melanoma and lung ctDNA using castPCR as an orthogonal method (S2 Fig). We detect the mutations with castPCR, but because of limited material, we were unable to run assays in duplicate or triplicate. However, we have previously performed rigorous validation of tumour sequencing with the Cancer Hotspot Panel on the Ion Torrent PGM^™^, and showed that Hotspot Panel Sequencing was successful in 97% of tumour samples (N = 351) and 100% concordant with known mutations (Hamblin et al., manuscript under review). This complete concordance indicated that the method was suitable for introduction into our routine NHS diagnostic service.

## Discussion

Blood was collected from patients with known melanomas for a retrospective pilot study, and we examined factors that may influence whether the confirmed mutations in the tumour were also detectable in plasma DNA. We only observe correlation between tumour and ctDNA variant allele frequency when the blood for ctDNA isolation was collected before treatment had started, or if collected less than one year after the tumour biopsy. With respect to treatment effects, although we did not do a time course study with successive samples taken from the same patient, we did observe a nearly complete absence of mutations in samples that were taken after treatment was started, which is most likely to be explained by the patients responding to targeted treatment. It is interesting to note that no further mutations were seen in all but one of these patient samples. Future longitudinal studies of ctDNA are warranted.

In the study with lung cancer patients, we specified in the standard operating procedure that blood draw for plasma DNA extraction occurred shortly (typically less than an hour) before tumour biopsy. This criterion was important because for several forms of cancer (e.g., breast, prostate, and lung), biopsies have been reported to increase the incidence of tumour cell seeding [20] and tumour cells in the circulation in animal models [21]. We demonstrate that ctDNA findings are in concordance with those found in the tumour for eight out of ten mutations detected. We also show it is possible to detect pathogenic mutations in ctDNA: While no *EGFR* mutations were detected in the 12 patients with lung cancer, a number of pathogenic variants were identified in oncogenic kinase genes such as *KRAS* and in tumour suppressor genes such as *TP53* or *PTEN*. In particular, we revealed pathogenic *TP53* mutations in ctDNA from all three squamous cell carcinoma patients, which has been shown to be the most commonly mutated gene in squamous lung cancer, as well as mutations in *CDKN2A* and *PTEN* [22]. Some of the identified mutations in lung adenocarcinoma affect treatment options in first or subsequent lines of systemic therapy (http://www.mycancergenome.org; https://ckb.jax.org/). For example, the *BRAF D594G* mutation results in BRAF inactivation and insensitivity to BRAF inhibitors. In the presence of activated *RAS*, inactivated BRAF can result in hyperactivation of *MEK* (*MAP2K1*), and thus MEK inhibitors may be effective in treating patients with *D594G* mutations, particularly when there is coexistent activation of *RAS*. The *N375S* mutation of the *MET* proto-oncogene confers resistance to MET targeted agents whereas *MET R988C* mutations in the juxta-membrane domain appear to have no effect on the capability of MET targeted agents to inhibit *cMET* phosphorylation. Other mutations (*JAK V722I*, *CTNNB1 G34V*, *SMAD4 R361H*) are rare in lung cancer and for which there is only preliminary or no evidence for targeted therapies. Although the lung cancer patients were not selected for specific criteria and the sample size was small, the proportions present of each type are similar to Cancer Research UK statistics for lung cancer incidence by morphology, which on their website are 87% non-small cell lung cancer (NSCLC, adenocarcinoma or squamous cell), 12% small cell, and 1% carcinoid (http://www.cancerresearchuk.org/health-professional/cancer-statistics/statistics-by-cancer-type/lung-cancer/incidence#heading-Four, accessed July 2016). Among our patients, we observe ten out of twelve (83%) NSCLC, one out of twelve (8%) small cell, and one out of twelve (8%) carcinoid tumour. Therefore our data has good representation of the different types of lung cancer. Others have found that targeted sequencing with the cancer hotspot panel is useful for advanced non-small cell lung cancer [23] and metastatic disease in a variety of tumour types [8]. We extend these results to using the hotspot panel to measure factors affecting mutation detection in melanoma ctDNA and evaluating use in initial diagnosis in lung cancer.

Collectively, these findings support using mutation analysis in ctDNA to provide a tumour profile helpful for diagnostic, predictive and prognostic analysis that does not require invasive procedures. Furthermore, the use of ctDNA also has potential for significant health economic, safety, and logistic benefits, whether as a means of obtaining repeat “liquid biopsies” from patients who are on treatment to allow monitoring of their mutanome, providing an early indication of whether or not patients are responding to targeted treatment, or after relapse of disease (where the standard of care is to undertake re-biopsy of the tumour to determine the presence of new mutations that may be actionable). Targeted therapies are costly and early identification of non-response (prior to symptomatic relapse) could save significant drug costs, as well as prevent unnecessary side effects. Panel ctDNA sequencing on the Ion Torrent PGM^™^ costs between £300–740 per sample, whereas transbronchial needle aspiration (TBNA) and endobronchial ultrasound (EBUS) cost £1365 in 2011, excluding tumour sequencing costs (NICE lung cancer costing report CG121, 2011). Lung cancers are often inaccessible to a bronchoscopically-guided tumour sampling, necessitating CT-guided biopsy that has inherent risks such as pneumothorax. Even in experienced hands, the amount of tumour material obtained by endobronchial or EBUS sampling may be insufficient or unsuitable for analysis. Further, these procedures are costly in terms of human resource, equipment and consumables. Our data suggest that sequencing plasma DNA would be a safer, cost effective yet just as informative a method to employ. Our findings also support the undertaking of further prospective studies of sequential ctDNA and tumour sequencing for patients throughout their treatment pathway. Identification of early disease progression or relapse, assessment of response and use within a screening programme are all potential applications of this relatively simple procedure.

In summary, we reveal two factors that influence concordance of mutation detection between primary tumour and ctDNA sequencing in melanoma: treatment status at time of blood sampling and time lag between sampling of the primary tumour and blood for ctDNA extraction. The findings provide evidence to support the use of plasma DNA sequencing to assess effectiveness of treatment, monitor cancer patients in remission, and provide an early indication of emerging mutations that could be amenable to targeted therapy. Additionally we find good but not perfect (80%) concordance of mutations in lung diagnostic biopsy and contemporaneous ctDNA, suggesting that liquid biopsy analysis could be a valuable first-line approach to confirm the diagnosis of lung cancer, especially when the tumour is inaccessible or when biopsy poses significant risk to the patient.

## Supporting Information

S1 Fig Spiked-in mutation positive control results showing the true spiked-in allele vs. false positives (noise) for S1 File sensitivity study. (TIF)

S2 Fig Comparison of ctDNA results for Ion Ampliseq sequencing with castPCR. We chose castPCR as an orthogonal platform to attempt validation of the Ion Ampliseq sequencing results. Nine castPCR assays were available to assess mutations in melanoma and lung cancer patients who had plasma DNA available for validation (less than the recommended 15–20ng DNA/well for the castPCR assay; assays were run in singlicate). X-axis, patient number and mutation tested; y-axis, percentage mutation. (A) Melanoma patient samples, (B) Lung patient samples. KIT M541L is a UCSC common polymorphism and not included in Table 1, but was assayed for technical validation.
(PDF)

S1 File Sensitivity study with spiked-in mutation-positive controls. (DOCX)

S1 Table Merged allele counts for spiked-in control kits for S1 File sensitivity study. (XLSX)

S2 Table Melanoma ctDNA sequencing pilot study. Blood samples were taken from melanoma patients who were known to have cancer (some patients were sampled pre-treatment, some were post-treatment). Tumour and plasma DNA were sequenced using Ampliseq Cancer Hotspot Panel (8–10 samples per 318 chip), and variant results were compared. For patients 9–14, ctDNA was also sequenced at higher coverage (using 1 ctDNA:gDNA paired sample per 318 chip).
(DOCX)

S3 Table Plasma DNA sequencing in non-cancer patients. Blood samples were drawn from patients prior to bronchoscopy. These patients were subsequently found to be negative for cancer. Plasma DNA and genomic DNA from each were sequenced using Ampliseq Cancer Hotspot Panel v2.
(XLSX)
