# Supplementary figures and images for: Targeted Next-Generation Sequencing of Plasma DNA from Cancer Patients: Factors Influencing Consistency with Tumour DNA and Prospective Investigation of Its Utility for Diagnosis
Source: PLoS One. 2016 Sep 14;11(9):e0162809. doi: 10.1371/journal.pone.0162809 (PMC5023174; doi:10.1371/journal.pone.0162809)

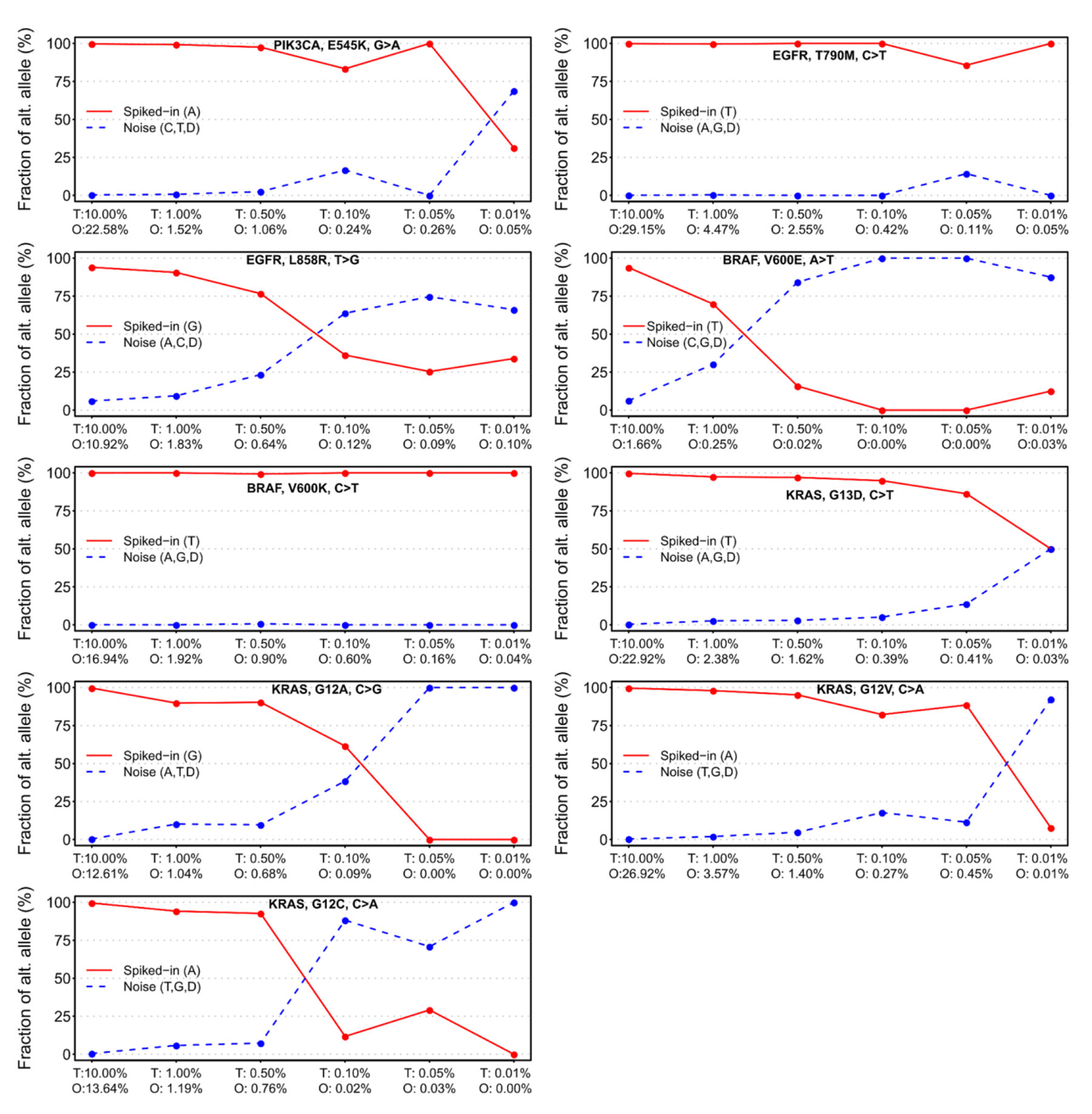

Supplement: S1 Fig — (TIF) [file pone.0162809.s001.tif]

S2 Fig

A. Melanoma

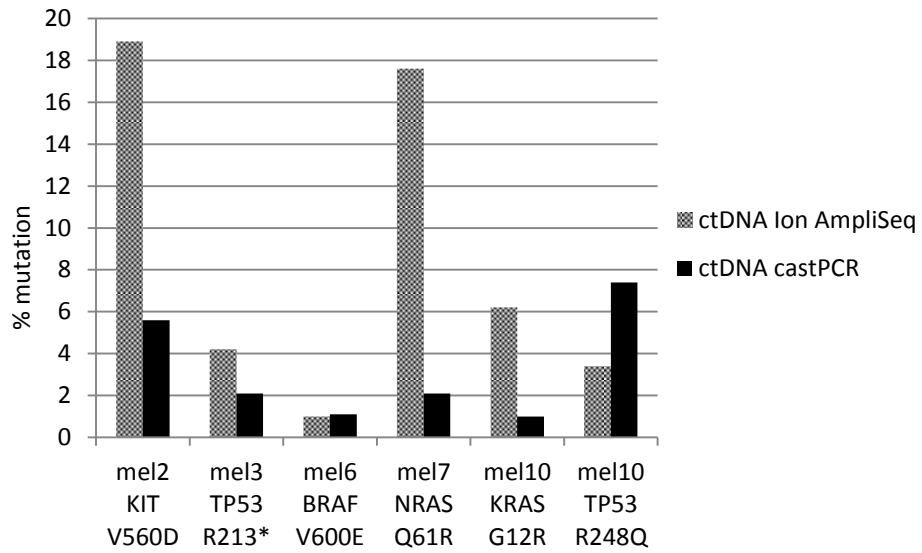

B. Lung

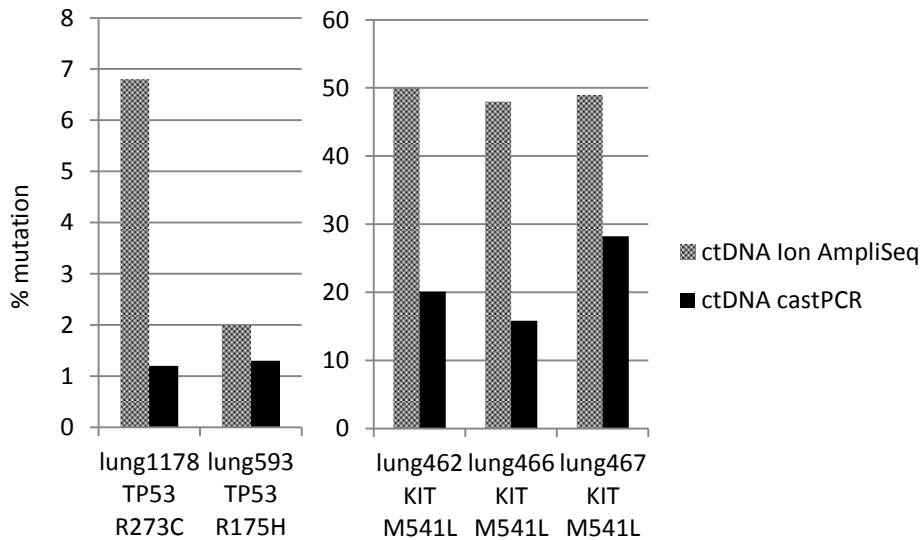

Supplement: S2 Fig — We chose castPCR as an orthogonal platform to attempt validation of the Ion Ampliseq sequencing results. Nine castPCR assays were available to assess mutations in melanoma and lung cancer patients who had plasma DNA available for validation (less than the recommended 15–20ng DNA/well for the castPCR assay; assays were run in singlicate). X-axis, patient number and mutation tested; y-axis, percentage mutation. (A) Melanoma patient samples, (B) Lung patient samples. KIT M541L is a UCSC common polymorphism and not included in Table 1, but was assayed for technical validation. (PDF) [file pone.0162809.s002.pdf]
