# Supplementary material for: Targeted Next-Generation Sequencing of Plasma DNA from Cancer Patients: Factors Influencing Consistency with Tumour DNA and Prospective Investigation of Its Utility for Diagnosis
Source: PLoS One. 2016 Sep 14;11(9):e0162809. doi: 10.1371/journal.pone.0162809 (PMC5023174; doi:10.1371/journal.pone.0162809)
Supplement: S1 File — (DOCX) [file pone.0162809.s003.docx]

**S1 File. Sensitivity study using spiked-in mutation-positive controls**

In this supporting study we looked at sensitivity of cancer hotspot panel sequencing as an aspect of next-generation sequencing in circulating tumour DNA (ctDNA). We used a dilution series of spiked-in mutation-positive DNA ranging from 10% to 0.01% concentration to simulate the frequently low levels of ctDNA in the blood.

# Method

## Spiked-in mutation-positive controls

Four positive control kits were prepared using DNA from three sources: 1) Four *KRAS* mutant cell lines, 2) One buffy coat from a healthy blood donor and 3) Six different plasmids. The *KRAS* mutant cell lines were sequenced using the Ion AmpliSeq Cancer Hotspot Panel v2. Genomic DNA (gDNA) from the buffy coat was extracted using iPrep PureLink gDNA Blood Kit (Life Technologies). The buffy coat gDNA tested negative for *KRAS*, *EGFR* and *BRAF* mutations. The cell line and plasmid DNA were used to spike-in mutations to the buffy coat gDNA using equal copies, resulting in a 50:50 mutant:wild-type ratio. For example, in Kit A, the *KRAS G12A* mutation and the *EGFR L747_S752* mutation were present in approximately equal amounts as the wild-type alleles. Four different mixes of mutations were made, and samples serially diluted with genomic DNA from 50% stocks to 10%, 1%, 0.5%, 0.1%, 0.05% and 0.01% (Table). AmpliSeq sequencing was performed with 10ng input DNA for all samples, described below. In order to determine the background level of variants present in these samples, we further sequenced each individual mutation-positive DNA component (from plasmid or cell line) and blood donor DNA, barcoded separately.

Table. Spiked mutation-positive control kit details.

| (A) Spiked-in kit composition | |  |  |  |
| --- | --- | --- | --- | --- |
|  | **Kit A** | **Kit B** | **Kit C** | **Kit D** |
| **Component** |  |  |  |  |
| wild-type gDNA | DNA extracted from buffy coat blood donation | | | |
| cell line DNA | **KRAS p.G12A** | **KRAS p.G12C** | **KRAS p.G12V** | **KRAS p.G13D** |
|  | TP53 p.A159D | FBXW7 p.R479Q | PDGFRA p.V824V | **PIK3CA p.E545K** |
|  |  | HRAS p.H27H | KIT p.M541L | SMO p.T640A |
|  |  | TP53 (chr17:7577538 C>T) | HRAS p.H27H | HRAS p.H27H |
|  |  |  | TP53 p.R273H | TP53 p.S241F |
|  |  |  | SMAD4 c.955+5G>C (intronic) | SMARCB1 c.1119-41G>A (intronic) |
| plasmid DNA | **EGFR L747_S752** | **EGFR T790M** | **EGFR L858R** | **EGFR L747_E749** |
| plasmid DNA |  | **BRAF V600K** | **BRAF V600E** |  |
|  |  |  |  |  |
| (B) Mutant allele concentration in each tube. | | |  |  |
|  | **Kit A** | **Kit B** | **Kit C** | **Kit D** |
| **Tube 1** | 0.50% | 0.10% | 0.05% | 0.05% |
| **Tube 2** | 0.05% | 1% | 0.50% | 0.01% |
| **Tube 3** | 0.01% | 0.50% | 1% | 0.10% |
| **Tube 4** | 0.10% | 0.05% | 0.01% | 1% |
| **Tube 5** | 1% | 0.01% | 0.10% | 0.50% |
| **Tube 6** | 10% | 10% | 10% | 10% |

(A) Spiked-in kit composition. The control kits were prepared by adding equal copies of mutation-positive cell line and plasmid DNA to wild-type genomic DNA (gDNA) from a blood donor, resulting in a 50:50 mixture of mutant:wild-type DNA. Hotspot mutations are shown in bold-face type. The TP53 (chr17:7577538 C>T) mutation has multiple coding sequence and amino acid nomenclature. Four kits with different mixes of mutations were made, as listed. (B) Mutant allele concentration in each tube. The samples were serially diluted from the 50% stocks with wild-type DNA to 10%, 1%, 0.5%, 0.1%, 0.05% and 0.01%, and arranged in random order for testing (tube 6 is always 10%).

## Sequence analysis

Because the Torrent Variant Caller (TVC) prior to version v4.4 was not optimised to detect variants below 1%, allelic frequencies were inferred from counts of reads containing the spiked-in mutations. Deletions were estimated by counting the reads carrying a deletion at the genomic midpoint of the given deletion. Reads were not filtered to avoid considerably distorting the allele frequencies of the highly diluted samples. This estimation of allelic frequency did not benefit from the statistical model or raw signal processing algorithm that was present in TVC, but it provided a reasonable estimate in the absence of a more sophisticated tool.

# Results

**Detection of known mutations in spiked-in mutation-positive control kits**

DNA carrying known mutations was spiked into control blood donor DNA, serially diluted and subsequently sequenced on the PGM™ using Cancer Hotspot Panel v.2 (CHPv2). By tallying the counts of reads containing the spiked-in mutations from the BAM files, we generated an initial result for all mutation-positive samples (S1 Table). We confirmed that the blood donor genomic DNA did not carry any hotspot or pathogenic mutations covered by the panel (S1 Table). We plotted the fraction of spiked-in alleles and other alternative alleles (“noise”) that we observed at each dilution, and used this to infer the sensitivity of this analysis (S1 Fig). Because the serial dilutions were made from the 10% tube, when this tube was imprecise the rest of the dilution series was shifted, resulting in observed allele frequencies that are different to the intended spiked-in concentration (O = observed in S1 Fig).

For all of the different hotspot mutations, the spiked-in alternative allele represents the vast majority of the alternative alleles detected in the 10% and 1% dilution tubes (S1 Fig). For five of the mutations (*PIK3CA E545K, EGFR T790M, BRAF V600K, KRAS G12V*, and *KRAS G13D)*, the spiked-in allele was the main alternative allele down to the 0.1% dilution (S1 Fig and S1 Table). The additional alternative alleles may have arisen due to inaccuracy at the dilution or library preparation stage or due to sequencing error. In some cases we observed the other alternative alleles at frequencies between 0.1 and 0.5%, which suggested that use of the Ion Torrent Variant Caller, which has a detection limit of 0.5%, would be suitable for routine analysis. Further sensitivity experiments could be performed using fragmented DNA to better simulate plasma DNA.

S1 Table caption. Merged allele counts for spiked-in control kits for S1 File sensitivity study. Raw allele counts of hotspot mutations sequenced with Ampliseq Cancer Hotspot Panel v2 are given for the different concentration tubes in each control kit (A1-A6, B1-B6, etc., composition described in Table 1), as well as for the individual components of the kits (Indiv-comp_wt-blood-gDNA, Indiv-comp-cell line, or Indiv-comp-plasmid). Blue shaded data indicate hotspot mutations that were spiked into a given kit. ‘Spiked-in AF [%]’ is the mutant allele frequency that was spiked into the tube; ‘Corrected AF [%]’ is the corrected concentration, calculated as a dilution of the measured mutant allele frequency in tube 6 (10%). The “diff to expected” column W is the difference between the corrected concentration (column C) and the measured mutant allele frequency (alternate allele %).

S1 Fig caption. Spiked-in mutation positive control results showing the true spiked-in allele vs. false positives (noise) for S1 File sensitivity study. For each hotspot mutation dilution series, the observed fraction of the spiked-in allele is plotted as a red solid line, and the fraction of other alleles (“noise”) is a blue dotted line (alternative alleles are in brackets; D = deletion). The x-axis is the spiked-in allele frequency concentration of the dilution series, from 10% down to 0.01% (T = theoretical spiked-in tube concentration), and below this are the actual allele frequencies observed by sequencing (O = observed). Because the serial dilutions were made from the 10% tube, when this tube was imprecise the rest of the dilution series was shifted. The y-axis shows the fraction of the alternative allele (spiked-in or noise), normalised to 100%. In other words the y-axis shows for each tube, what percentage of the alternative allele pool was the allele we spiked-in, and what percentage were noise alleles.
