# Supplementary material for: Targeted Next-Generation Sequencing of Plasma DNA from Cancer Patients: Factors Influencing Consistency with Tumour DNA and Prospective Investigation of Its Utility for Diagnosis
Source: PLoS One. 2016 Sep 14;11(9):e0162809. doi: 10.1371/journal.pone.0162809 (PMC5023174; doi:10.1371/journal.pone.0162809)
Supplement: S2 Table — Blood samples were taken from melanoma patients who were known to have cancer (some patients were sampled pre-treatment, some were post-treatment). Tumour and plasma DNA were sequenced using Ampliseq Cancer Hotspot Panel (8–10 samples per 318 chip), and variant results were compared. For patients 9–14, ctDNA was also sequenced at higher coverage (using 1 ctDNA:gDNA paired sample per 318 chip). (DOCX) [file pone.0162809.s005.docx]

S2 Table. Melanoma ctDNA sequencing retrospective pilot study results. Blood samples were taken from melanoma patients who were known to have cancer (some patients were sampled pre-treatment, some were post-treatment). Tumour and plasma DNA were sequenced using Ampliseq Cancer Hotspot Panel (8-10 samples per 318 chip), and variant results were compared. For patients 9-14, ctDNA was also sequenced at higher coverage (using 1 ctDNA:gDNA paired sample per 318 chip).

| **Patient no.** | **Sample** | **Sample Date** | **Time dif (days btn tumour and blood sampling)** | **Variant** | **VAF (%)** | **Treatment notes** | **Pre or post treatment blood sampling** |
| --- | --- | --- | --- | --- | --- | --- | --- |
| 1 | Tumour biopsy | 01/10/2009 | 1206 | BRAF V600R | 35 | Started Vemurafenib (BRAF inhibitor) on 09/05/2013, then Ipilimumab (immune system activator), which finished 17/10/2013. | pre- treatment |
|  | ctDNA | 19/01/2013 |  | BRAF V600R | 7 |  |  |
| 2 | Tumour biopsy | 19/04/2013 | 62 | c-KIT V560D | 80 | Nilotinib (c-KIT inhibitor) started 03/07/2013. | pre- treatment |
|  | ctDNA | 20/06/2013 |  | c-KIT V560D | 19 |  |  |
| 3 | Tumour biopsy | 09/03/2012 | 594 | TP53 R213* | 61 | Started chemotherapy on 14/11/2013. | pre- treatment |
|  | ctDNA | 24/10/2013 |  | TP53 R213* | 4 |  |  |
| 4 | Tumour biopsy | 20/02/2013 | 36 | c-KIT N655T | 36 | Started chemotherapy on 19/04/2013. | pre- treatment |
|  | ctDNA | 28/03/2013 |  | c-KIT N655T | 1.1 |  |  |
| 5 | Tumour biopsy | 15/05/2012 | 542 | BRAF V600E | 14 | Stage 3B melanoma excised in 2012. Started Vemurafenib 19/11/2013. | pre- treatment |
|  | ctDNA | 07/11/2013 |  | BRAF V600E | 1 |  |  |
| 6 | Tumour biopsy | 30/09/2013 | 31 | NRAS Q61R | 79 | Has brain metastases, started RADVAN study (whole brain radiotherapy and Vandetanib (kinase inhibitor)/placebo) on 14/11/2013. | pre- treatment |
|  | ctDNA | 31/10/2013 |  | NRAS Q61R | 18 |  |  |
| 7 | Tumour biopsy | 25/08/2011 | 588 | BRAF V600E | 25 | Started Vemurafenib 2/5/2013. | pre- treatment |
|  | ctDNA (low coverage) | 04/04/2013 |  | negative | - |  |  |
|  | ctDNA (high coverage) | 04/04/2013 |  | BRAF V600E | 0.8 |  |  |
| 8 | Tumour biopsy | 26/02/2013 | 93 | BRAF V600E | 49 | Started Vemurafenib on 30/5/2013. | pre- treatment |
|  | ctDNA (low coverage) | 30/05/2013 |  | negative | - |  |  |
|  | ctDNA (high coverage) | 30/05/2013 |  | BRAF V600E | 0.7 |  |  |
| 9 | Tumour biopsy | not available | na | BRAF V600E | not avail. | ctDNA seq detects mutation seen in tumour and one additional. Started Vemurafenib on 22/8/2013. | pre- treatment |
|  | ctDNA | 22/08/2013 |  | BRAF V600E | 14 |  |  |
|  |  |  |  | CTNNB1 T41A | 6 |  |  |
| 10 | Tumour biopsy | 18/07/2011 | 633 | BRAF V600E | 36 | Surgical treatment followed by Vemurafenib, 21/5/2013 start. | pre- treatment |
|  | ctDNA | 11/04/2013 |  | negative | - |  |  |
| 11 | Tumour biopsy | 13/10/2010 | 981 | negative |  | Died Jul 2013. | pre- treatment |
|  | ctDNA | 20/06/2013 |  | KRAS G12R | 6 |  |  |
|  |  |  |  | TP53 R248Q | 3.4 |  |  |
| 12 | Tumour biopsy | 25/10/2011 | 604 | BRAF V600E | 45 | Treated with Dacarbazine, then Ipilimumab. Started Dabrafenib (BRAF inhibitor) in Sept 2012. | post- treatment |
|  | ctDNA | 20/06/2013 |  | BRAF V600E | 50 |  |  |
| 13 | Tumour biopsy | 26/04/2006 | 2559 | BRAF V600E | 27 | Treated with Vemurafenib Jan-Jun 2013, then Ipilimumab Jun-Aug 2013. Died Aug 2013 due to progressive disease. | post- treatment |
|  | ctDNA (low coverage) | 28/03/2013 |  | negative | - |  |  |
|  | ctDNA (high coverage) | 28/03/2013 |  | negative | - |  |  |
| 14 | Tumour biopsy | 23/03/2012 | 559 | BRAF V600E | 33 | Dabrafenib (BRAF inhib) and Trametinib (MEK inhib) started Jul 2012 (Combi-D study), stopped Apr 2013 due to toxicity. Died Nov 2013 due to progressive disease. | post- treatment |
|  |  |  |  | TP53 S241F | 53 |  |  |
|  | ctDNA (low coverage) | 03/10/2013 |  | negative | - |  |  |
|  | ctDNA (high coverage) |  |  | negative | - |  |  |
| 15 | Tumour biopsy | 22/08/2012 | 267 | BRAF V600E | 51 | Started Vemurafenib 14/3/2013. | post- treatment |
|  | ctDNA (low coverage) | 16/05/2013 |  | negative | - |  |  |
|  | ctDNA (high coverage) |  |  | negative | - |  |  |
| 16 | Tumour biopsy | 06/07/2012 | 265 | NRAS Q22K | 44 | Started Dacarbazine chemotherapy 14/3/2013, stopped due to toxicity. Died May 2013 due to progressive disease. | post- treatment |
|  | ctDNA (low coverage) | 28/03/2013 |  | negative | - |  |  |
|  | ctDNA (high coverage) |  |  | negative | - |  |  |
| 17 | Tumour biopsy | 09/05/2012 | 253 | BRAF V600E | 49 | Started Vemurafenib June 2012. | post- treatment |
|  | ctDNA | 17/01/2013 |  | negative | - |  |  |
| 18 | Tumour biopsy | not available | na | BRAF V600E | not avail. | Treated with Vemurafenib, then IMCgp100 (immune-modulating agent), then Ipilimumab, completed Dec 2012. | post- treatment |
|  | ctDNA | 28/03/2013 |  | negative | - |  |  |
| 19 | Tumour biopsy | 14/01/2013 | 87 | TP53 Q60X | 63 | Melanoma removed surgically Nov 2012. | post- treatment |
|  | ctDNA | 11/04/2013 |  | negative | - |  |  |
| 20 | Tumour biopsy | 06/12/2012 | 126 | BRAF V600E | 30 | Vemurafenib started Feb 2013, stopped Jan 2015 (because of progressive disease). Died Mar 2015. | post- treatment |
|  | ctDNA | 11/04/2013 |  | negative | - |  |  |
| 21 | Tumour biopsy | 09/11/2011 | 624 | NRAS Q61H | 71 | Melanoma removed Jun 2011, Jan-Mar 2012 treated in DOC-MEK study (stopped due to progressive disease), treated with Ipilimumab Jun-Aug 2012. On follow-up. | post- treatment |
|  |  |  |  | PTEN L325F | 60 |  |  |
|  | ctDNA | 25/07/2013 |  | negative | - |  |  |
